# Kidney-sparing immunotherapy for lynch syndrome-associated upper tract urothelial carcinoma following colon cancer: a case report

**DOI:** 10.3389/fonc.2026.1909587

**Published:** 2026-09-18

**Authors:** Haibiao Xie, Qifan Xie, Chunxiang Feng, Kun Wei, Fangqianyu Zhong, Jie Wen, Jiawei Gao, Xiaoyong Pu, Chujin Ye

**Affiliations:** 1 Department of Urology, Guangdong Provincial People's Hospital, Guangdong Academy of Medical Sciences, Southern Medical University, Guangzhou, China; 2 Department of Urology, Guangdong Second Traditional Chinese Medicine Hospital (Guangdong Research Institute of Traditional Chinese Medicine Engineering and Technology), Guangzhou, China; 3 Department of Urology, School of Medicine, South China University of Technology, Guangzhou, China

**Keywords:** colon cancer, immunotherapy, kidney-sparing treatment, lynch syndrome, microsatellite instability-high, mismatch repair deficiency, upper tract urothelial carcinoma

## Abstract

Lynch syndrome (LS) is a hereditary cancer predisposition syndrome associated with multiple malignancies, including colorectal cancer and upper tract urothelial carcinoma (UTUC). We report a 61-year-old woman who initially presented with ascending colon adenocarcinoma and underwent laparoscopic right hemicolectomy. Immunohistochemistry showed loss of MLH1 and PMS2 expression, suggesting deficient mismatch repair. Ten months later, she developed a right renal pelvic mass, which was confirmed by ureteroscopic biopsy as invasive high-grade urothelial carcinoma with squamous differentiation rather than metastatic colon cancer. The urothelial tumor also showed an abnormal MLH1/PMS2 immunophenotype. Subsequent molecular evaluation included plasma cell-free DNA profiling for somatic genomic characterization and peripheral-blood germline analysis for hereditary cancer assessment. Germline testing identified a heterozygous pathogenic variant in NM_000249.4(*MLH1*):c.503dup (p.Asn168fs), establishing the diagnosis of Lynch syndrome. Because the patient strongly wished to preserve renal function, she received single-agent toripalimab at 240 mg every 3 weeks instead of radical nephroureterectomy. Contrast-enhanced CT after three cycles showed tumor reduction, and after six cycles demonstrated further marked regression of the right renal pelvic and upper ureteral lesion, with only residual focal thickening of the renal pelvic wall. At the last follow-up in April 2026, the patient remained clinically stable without evidence of colorectal cancer recurrence or progression of the UTUC. This case highlights the importance of tissue confirmation of newly developed lesions and timely etiologic evaluation of abnormal MMR findings in patients with suspected LS. It adds to emerging evidence that PD-1 blockade may have a kidney-preserving role in carefully selected patients with dMMR LS-associated UTUC, although longer radiographic, endoscopic, cytologic, and histologic surveillance is required.

## Introduction

Lynch syndrome (LS) is an autosomal dominant hereditary cancer syndrome caused by pathogenic germline variants in DNA mismatch repair (MMR) genes *MLH1*, *MSH2*, *MSH6*, and *PMS2*, or by deletions involving *EPCAM* that result in epigenetic silencing of *MSH2* (1). Rarely, a Lynch phenotype may result from constitutional epigenetic silencing of an MMR gene, particularly *MLH1*. Such constitutional epimutations may arise *de novo* or show non-Mendelian inheritance patterns (2). Loss of MMR function leads to microsatellite instability (MSI) and increases the risk of multiple malignancies (3). Although colorectal and endometrial cancers are the most widely recognized LS-associated malignancies, the disease spectrum also includes cancers of the stomach, small intestine, pancreas, ovary, biliary tract, brain, skin, and urinary tract (4).

Among urologic malignancies, upper tract urothelial carcinoma (UTUC) is considered one of the most clinically important LS-associated tumors (5). A 2026 systematic review and meta-analysis including 2, 378 patients with UTUC estimated a pooled prevalence of pathogenic germline MMR variants of 3.2% (95% CI, 2.1%–4.4%), with MSH2 representing the most frequently affected MMR gene (6). In parallel, a systematic review of urothelial carcinoma reported pooled prevalences of dMMR and MSI-high (MSI-H) of approximately 8.95% and 8.36%, respectively, in UTUC, substantially higher than those observed in bladder urothelial carcinoma (7). These findings support consideration of LS in patients with UTUC, particularly in those with another LS-spectrum malignancy or an abnormal MMR phenotype, even when the family history is noncontributory.

The dMMR/MSI-H phenotype also predicts sensitivity to immune checkpoint blockade targeting programmed cell death protein 1 (PD-1) or its ligand (8–10). Evidence specifically addressing immunotherapy in molecularly selected UTUC has recently strengthened. Moussa et al. retrospectively evaluated 24 patients with advanced dMMR/MSI-H UTUC treated with single-agent immune checkpoint inhibitors and reported a confirmed objective response rate of 83%, including 16 complete responses; genetically confirmed LS was present in 16 patients (67%) (11). These results provide important cohort-level evidence for the sensitivity of dMMR/MSI-H UTUC to immune checkpoint inhibition, although the retrospective, single-center design remains hypothesis-generating and does not establish immunotherapy as a standard kidney-preserving alternative to radical nephroureterectomy.

Here, we report a patient with genetically confirmed *MLH1*-associated LS who developed metachronous primary malignancies of the ascending colon and renal pelvis. This case complements the emerging cohort-level evidence by documenting histopathologic confirmation of a metachronous second primary tumor and the use of single-agent toripalimab with an explicit kidney-preservation intent after the patient declined radical nephroureterectomy. The case therefore provides a detailed example of molecularly guided, organ-preservation-oriented management while recognizing that the oncologic durability of this strategy requires longer-term evaluation.

## Case reports

A 61-year-old woman presented to our hospital in January 2025 with a two-month history of right lower abdominal pain and abdominal distension. A structured personal and three-generation family history was obtained. No malignant tumors were reported among her first-, second-, or third-degree relatives. The patient had no personal history of endometrial carcinoma or its recognized precursor lesions, including a typical endometrial hyperplasia. Colonoscopy revealed an obstructing cauliflower-like mass in the ascending colon, approximately 70 cm from the anal verge. Contrast-enhanced abdominal computed tomography (CT) suggested ascending colon cancer with suspected multiple regional lymph node metastases; the mass measured 100 × 63 mm (**Figures 1A, B**). Serum carcinoembryonic antigen and carbohydrate antigen 19–9 levels were elevated to 9.39 ng/mL (reference range, 0–5 ng/mL) and 222 U/mL (reference range, 0–30 U/mL), respectively.

**Figure 1:**
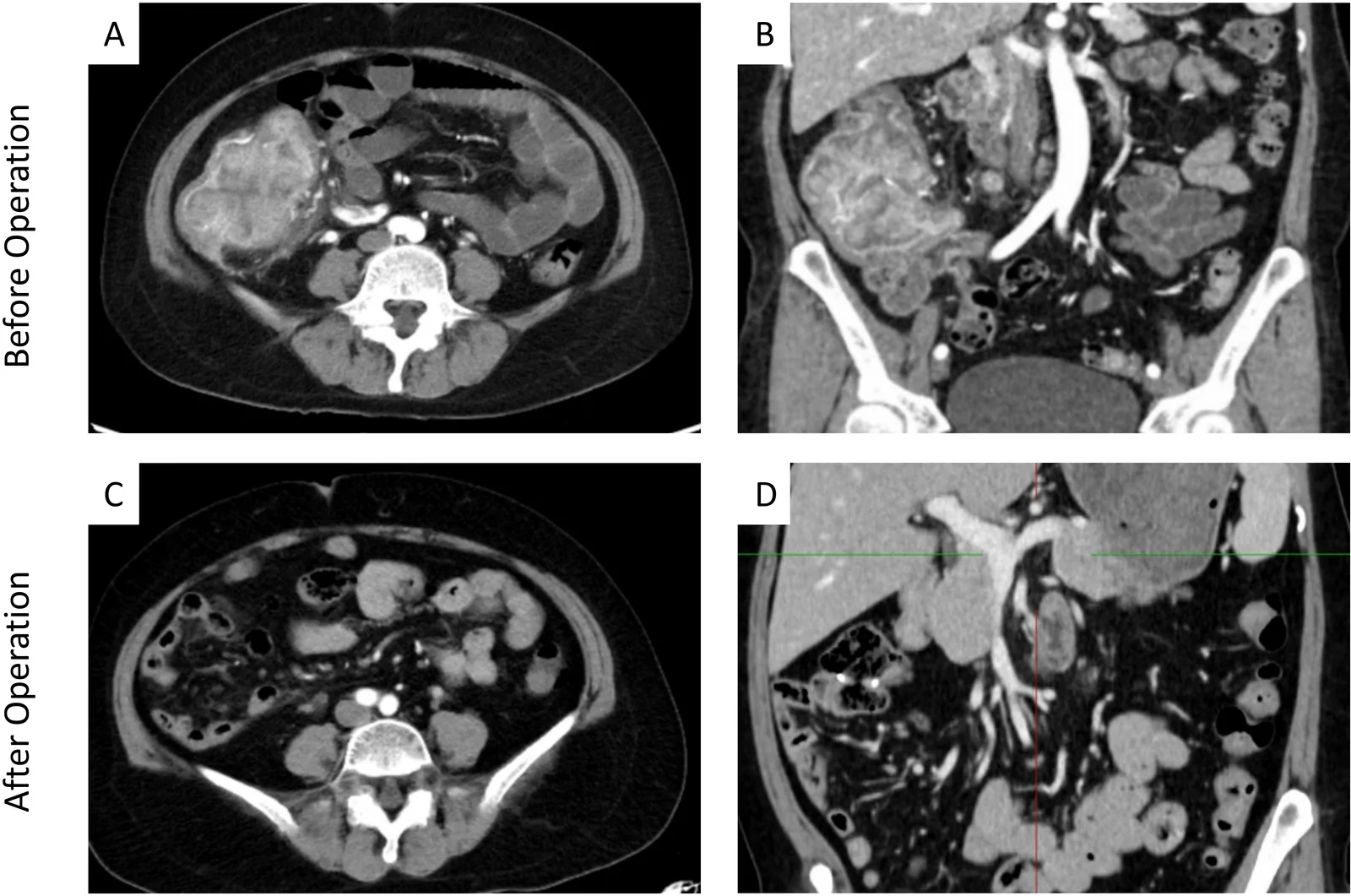
Radiological findings of ascending colon cancer before and after surgery. **(A, B)** Preoperative contrast-enhanced CT images showing a large mass in the ascending colon. **(C, D)** Postoperative CT images after laparoscopic right hemicolectomy showing postoperative changes without obvious local recurrence.

On January 9, 2025, the patient underwent laparoscopic right hemicolectomy with regional lymphadenectomy and ileocolic anastomosis. Histopathological examination of the resected specimen demonstrated moderately to poorly differentiated adenocarcinoma invading the subserosa (pT3N0, 0/24 lymph nodes), accompanied by prominent intratumoral lymphocytic infiltration. Immunohistochemistry (IHC) showed loss of MLH1 and PMS2 protein expression, whereas MSH2 and MSH6 were retained, indicating dMMR and raising suspicion of LS (**Figure 2**). Further molecular and genetic evaluation was recommended at that time; however, the patient declined additional testing. No adjuvant chemotherapy was administered, and the patient entered regular postoperative surveillance. During follow-up, no obvious evidence of recurrence was observed (**Figures 1C, D**).

**Figure 2:**
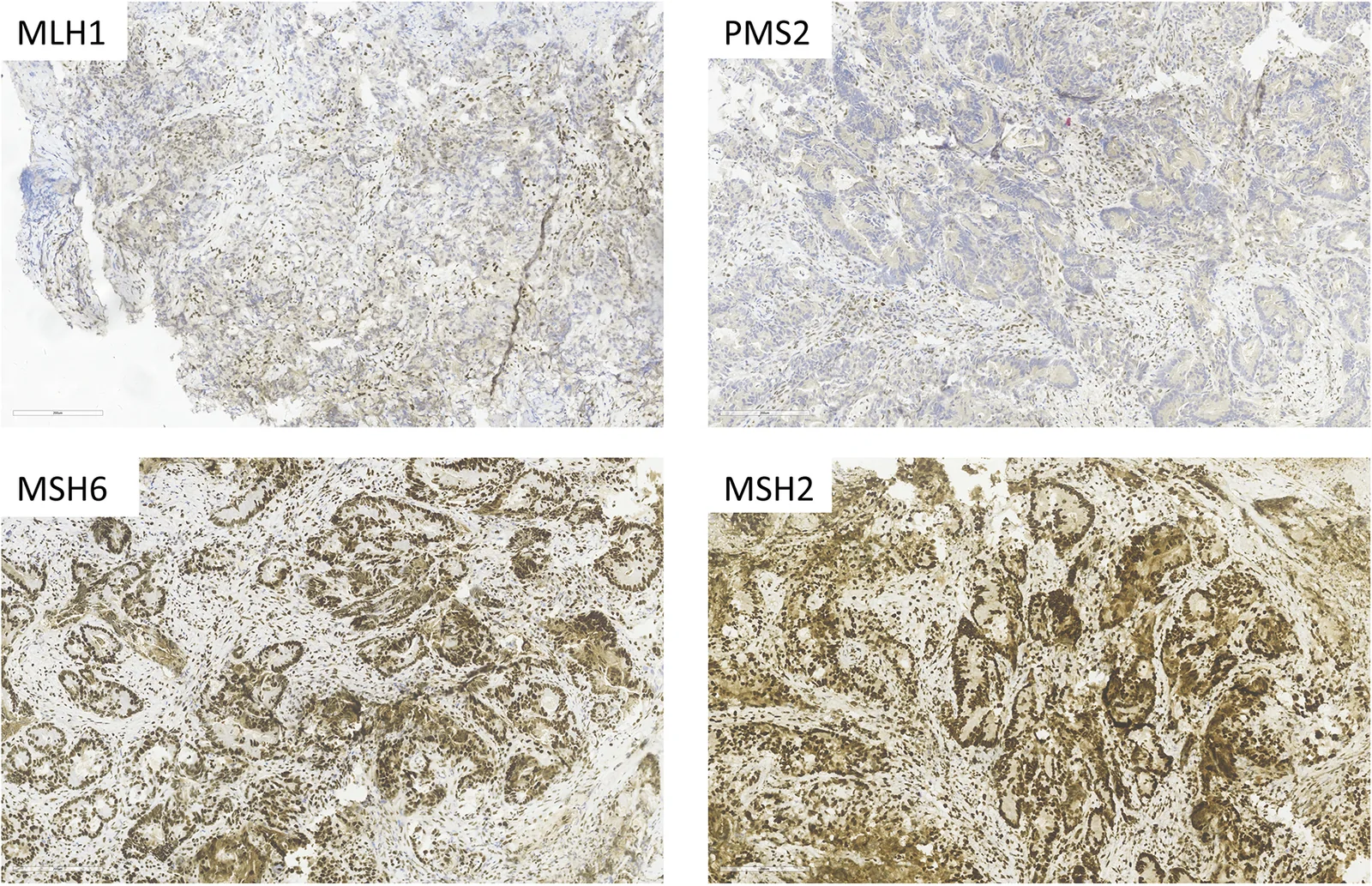
Immunohistochemical staining of mismatch repair proteins in the colon adenocarcinoma. Tumor cells showed loss of MLH1 and PMS2 expression, whereas MSH2 and MSH6 expression was retained.

Ten months later, in November 2025, the patient presented to the urology department with a two-month history of right flank pain. Positron emission tomography/computed tomography (PET/CT) revealed an irregular hypermetabolic soft-tissue mass (4.8 × 4.2 × 5.1 cm; SUVmax, 43.7) involving the right renal pelvis and the lower pole of the right kidney. Multiple hypermetabolic paracaval retroperitoneal lymph nodes located adjacent to the inferior vena cava were also identified (SUVmax, 9.3) (**Figures 3A, B**). No evidence of colon cancer recurrence was observed. Ureteroscopic biopsy of the renal pelvic mass was subsequently performed. Pathology confirmed invasive high-grade urothelial carcinoma with squamous differentiation, excluding metastasis from colon cancer. The urothelial tumor showed an abnormal MMR profile characterized by MLH1 loss with heterogeneous/partial PMS2 expression and retained MSH2/MSH6 expression (**Figure 4**). Following the diagnosis of the renal pelvic tumor, the patient agreed to further molecular evaluation. Molecular testing was performed for two purposes. Plasma cell-free DNA sequencing was performed as a tumor-associated somatic profiling assay to identify potentially actionable genomic alterations, whereas DNA extracted from the peripheral-blood sample was analyzed separately for germline variants associated with hereditary cancer predisposition. Plasma profiling was performed using a targeted-region probe-capture NGS assay on an Illumina sequencing platform with GRCh37/hg19 as the reference genome. Germline analysis of peripheral-blood DNA identified a heterozygous pathogenic variant in NM_000249.4(*MLH1*):c.503dup (p.Asn168fs), (**Table 1**) thereby establishing the diagnosis of *MLH1*-associated Lynch syndrome. The diagnosis was further supported clinically by the occurrence of two metachronous Lynch syndrome-associated primary tumors with abnormalities involving the MLH1/PMS2 mismatch-repair pathway. Cascade genetic testing was recommended for at-risk relatives; however, predictive testing was not performed because of financial constraints, and the carrier status of the patient’s relatives therefore remains unknown.

**Figure 3:**
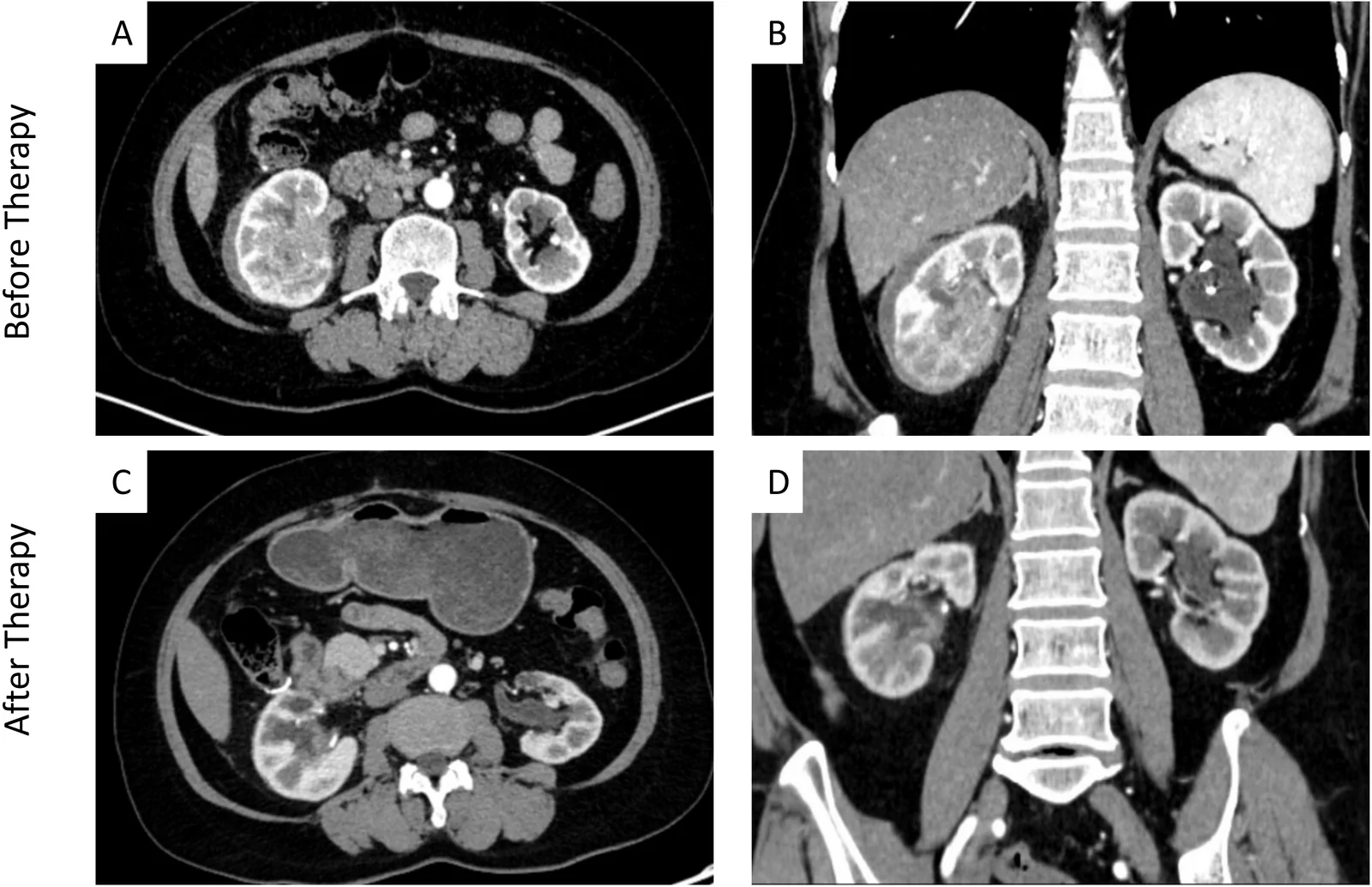
Radiological response of right renal pelvic upper tract urothelial carcinoma to immunotherapy. **(A, B)** Contrast-enhanced CT images before immunotherapy showing an irregular mass involving the right renal pelvis and lower pole of the right kidney. **(C, D)** Corresponding CT images obtained after six cycles of toripalimab show marked tumor regression, with only residual focal wall thickening remaining.

**Figure 4:**
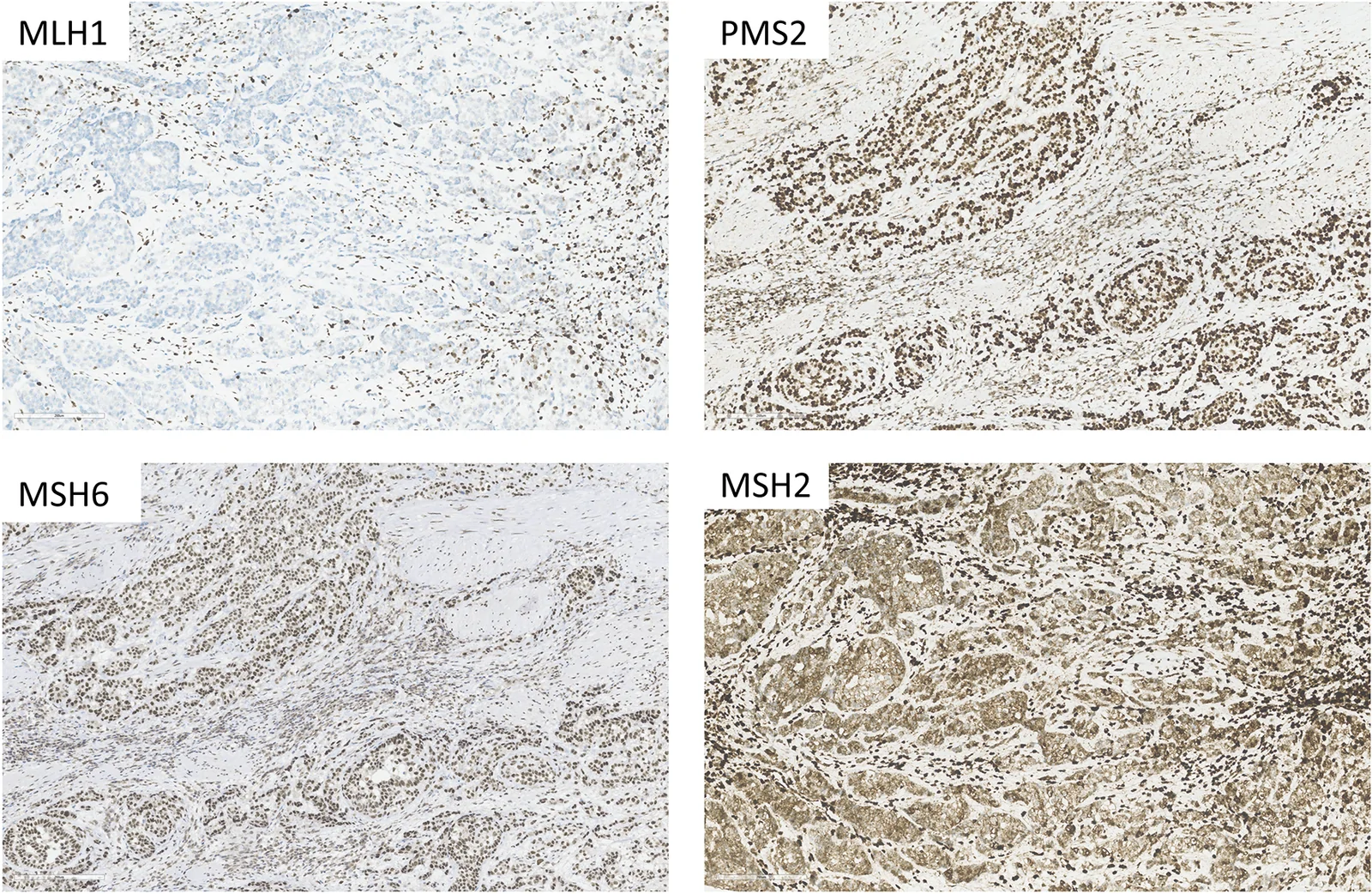
Immunohistochemical findings of mismatch repair proteins in the renal pelvic tumor. Immunohistochemical staining of the ureteroscopic biopsy specimen showed loss of MLH1 expression in tumor cells, with heterogeneous/partial PMS2 expression and retained MSH2 and MSH6 expression.

**Table 1:** Germline genetic testing result of the patient.

Gene	Variant	Variant type	Zygosity	ACMG classification	Associated syndrome	Inheritance pattern
*MLH1*	NM_000249.4:c.503dup (p.Asn168fs)	Germline frameshift variant	Heterozygous	Pathogenic	Lynch syndrome	Autosomal dominant

Because the patient strongly wished to preserve her kidney, systemic immunotherapy was initiated instead of radical nephroureterectomy. She received intravenous toripalimab at a dose of 240 mg every 3 weeks. After three cycles of toripalimab, contrast-enhanced CT demonstrated a clear reduction in the tumor involving the right renal pelvis and upper right ureter compared with baseline. After six cycles of treatment, follow-up contrast-enhanced CT demonstrated progressive and marked shrinkage of the right renal pelvic tumor, which ultimately regressed to residual focal wall thickening only (**Figures 3C, D**). Serial imaging therefore demonstrated a sustained radiographic response rather than complete radiographic disappearance of the lesion. At the last follow-up in April 2026, the patient remained clinically stable, with no evidence of colorectal cancer recurrence or clinical evidence of progression of the upper tract urothelial carcinoma. No severe immune-related adverse events were documented during treatment and follow-up.

## Discussion

This case describes genetically confirmed *MLH1*-associated LS with metachronous ascending colon adenocarcinoma and high-grade renal pelvic UTUC, followed by a radiographic partial response to single-agent toripalimab used with kidney-preserving intent. The colon tumor showed loss of MLH1 and PMS2 with retained MSH2/MSH6, whereas the renal pelvic tumor showed MLH1 loss with heterogeneous/partial PMS2 expression and retained MSH2/MSH6. Together with the pathogenic germline *MLH1* variant, these findings supported Lynch syndrome-associated metachronous primary tumors. The significance of this case lies not only in the occurrence of LS-associated metachronous double primary malignancies, but also in the fact that molecular tumor testing enabled recognition of the hereditary syndrome and guided a precise kidney-preserving therapeutic strategy.

The initial colorectal tumor showed combined MLH1/PMS2 protein loss. In colorectal cancer, this immunohistochemical pattern ordinarily prompts tumor-based reflex testing for *BRAF* V600E and/or *MLH1* promoter hypermethylation, because either finding strongly favors sporadic *MLH1* silencing rather than Lynch syndrome. Importantly, this colorectal cancer-specific triage approach is not an established method for determining the etiology of MMR deficiency in urothelial carcinoma (3, 12). At the initial colorectal cancer diagnosis, further molecular and genetic evaluation was recommended because of MLH1/PMS2 loss; however, the patient declined additional testing. Consequently, tumor-based *BRAF* V600E and *MLH1* promoter methylation testing and germline evaluation were not performed at that time. The renal pelvic urothelial carcinoma independently demonstrated an abnormal MMR immunophenotype, which further increased clinical suspicion of an underlying hereditary MMR defect. Definitive germline analysis of peripheral blood identified a heterozygous pathogenic variant in NM_000249.4(*MLH1*):c.503dup (p.Asn168fs), establishing the diagnosis of *MLH1*-associated Lynch syndrome. This molecular finding is particularly informative because the patient reported no family history of malignancy. A negative family history does not exclude Lynch syndrome, as hereditary cancer patterns may be obscured by limited family size, incomplete penetrance, unrecognized cancers in relatives, or a *de novo* pathogenic variant. Overall, this case highlights the importance of initiating etiologic evaluation when MLH1/PMS2 loss is first identified, irrespective of family history, to facilitate earlier recognition of Lynch syndrome and appropriate genetic counseling and surveillance.

The renal pelvic lesion was right-sided, ipsilateral to the prior ascending colon cancer, which initially raised concern for recurrence or metastatic spread. However, laterality alone was insufficient to establish a relationship between the two malignancies. The renal pelvic mass was anatomically separate from the previous colonic operative bed, and the suspected metastatic retroperitoneal lymph nodes were located in the paracaval region at the level of the kidney, anatomically distinct from the regional ileocolic/right-colic lymphatic drainage basin of the original ascending colon cancer. Most importantly, ureteroscopic biopsy demonstrated high-grade urothelial carcinoma with squamous differentiation rather than adenocarcinoma. Although the shared dMMR phenotype supported a common hereditary predisposition, it did not by itself establish a clonal relationship between the two tumors. Taken together, the distinct histopathologic features, site-specific anatomy and nodal distribution, absence of colorectal cancer recurrence, and identification of a pathogenic germline *MLH1* variant supported the diagnosis of metachronous primary malignancies.

With regard to treatment, radical nephroureterectomy remains the standard approach for high-risk UTUC. However, in patients with LS, treatment decisions should balance oncologic control with long-term preservation of renal function. Patients with LS have a lifelong risk of developing multiple primary tumors, and urinary tract malignancies may be bilateral or metachronous. Radical nephroureterectomy may substantially reduce renal functional reserve, thereby limiting future treatment options and affecting quality of life (13). Evidence supporting immunotherapy in molecularly selected UTUC has strengthened. Moussa et al. reported 24 patients with advanced dMMR/MSI-H UTUC treated with single-agent immune checkpoint blockade: 83% achieved a confirmed objective response, including 16 complete responses, and 67% had confirmed LS; however, the retrospective, single-center design was explicitly hypothesis-generating (11). The present case adds a detailed example of toripalimab use in an *MLH1* carrier with metachronous colorectal cancer and tissue-confirmed dMMR renal pelvic UTUC in the context of an explicit kidney-preservation goal.

The patient received intravenous toripalimab at a dose of 240 mg every 3 weeks. After three cycles, contrast-enhanced CT showed a clear reduction in the tumor involving the right renal pelvis and upper right ureter. After six cycles, further marked regression was observed, with the previously mass-forming lesion reduced predominantly to residual focal thickening of the renal pelvic wall. The patient achieved a sustained radiographic partial response without severe immune-related adverse events. This response is consistent with the biological sensitivity of dMMR tumors to PD-1/PD-L1 blockade (10). Le et al. demonstrated that MMR-deficient tumors harbor a high somatic mutational burden and may be more sensitive to PD-1 blockade because of an increased number of mutation-associated neoantigens (14). Subsequent studies have further established dMMR/MSI-H status as a pan-cancer predictive biomarker for immune checkpoint inhibitor efficacy (14, 15). In urothelial carcinoma, toripalimab has demonstrated antitumor activity with an acceptable safety profile. In POLARIS-03, toripalimab produced an objective response rate of approximately 26.5% with a manageable safety profile in previously treated advanced urothelial carcinoma (16), and a 2026 subgroup analysis demonstrated clinically meaningful activity in previously treated metastatic UTUC (17). In this patient, toripalimab was chosen on the basis of local availability, institutional experience, the strong biologic rationale provided by dMMR, and the patient’s preference to avoid nephroureterectomy. Dual PD-1/CTLA-4 blockade was considered as a potential alternative but was not selected because of concerns regarding treatment-related toxicity and the lack of evidence supporting its superiority in this specific clinical setting. Although the CheckMate 032 trial demonstrated higher response rates with nivolumab plus ipilimumab than with nivolumab monotherapy in previously treated metastatic urothelial carcinoma, the combination regimen, particularly the higher-ipilimumab-dose cohort, was associated with increased rates of grade 3–4 treatment-related adverse events (18). Similarly, a previous case report of bilateral UTUC suggested that neoadjuvant PD-1 blockade alone may be feasible for kidney sparing and avoidance of hemodialysis, although current evidence remains largely limited to case reports and retrospective studies (19). Therefore, the use of single-agent toripalimab in this patient represented a risk-adapted, individualized treatment decision.

Several limitations should be acknowledged. The follow-up duration is short, the treatment response was assessed radiographically without pathological confirmation, and residual focal wall thickening remains. Therefore, this single case cannot establish durable renal preservation, survival benefit, or equivalence to radical nephroureterectomy. Post-treatment endoscopic evaluation and close surveillance remain necessary, particularly for high-risk patients managed with kidney-sparing intent. In accordance with AUA guideline recommendations, we implemented intensified surveillance with upper-tract cross-sectional imaging every 3 months and cystoscopy with urinary cytology every 6 months (20). This case provides further support for the potential role of PD-1 blockade in selected dMMR/MSI-H UTUC patients seeking organ preservation, but does not justify replacing surgery as standard care. Prospective studies with longer follow-up and standardized response assessment are warranted.

## Conclusion

We report a patient with LS caused by a pathogenic germline *MLH1* variant who successively developed ascending colon adenocarcinoma and high-grade urothelial carcinoma of the renal pelvis as metachronous double primary malignancies. The case emphasizes prompt application of the MLH1/PMS2-loss diagnostic algorithm, detailed personal and family history assessment, and tissue confirmation of any new lesion. A partial radiographic response to single-agent toripalimab allowed renal preservation during the reported follow-up, but the observation remains preliminary. This case supports further study of molecularly selected, kidney-preserving immunotherapy strategies in dMMR/MSI-H UTUC and underscores the need for rigorous endoscopic and imaging surveillance.

## Data Availability

The raw data supporting the conclusions of this article will be made available by the authors, without undue reservation.
